# Differential Responses of Urinary Epinephrine and Norepinephrine to 24-h Shift-Work Stressor in Physicians

**DOI:** 10.3389/fendo.2020.572461

**Published:** 2020-09-23

**Authors:** Claudia Boettcher, Grit Sommer, Mirko Peitzsch, Klaus-Peter Zimmer, Graeme Eisenhofer, Stefan A. Wudy

**Affiliations:** ^1^University Children's Hospital, Paediatric Endocrinology & Diabetology, University of Berne, Berne, Switzerland; ^2^Division of Paediatric Endocrinology & Diabetology, Department of General Paediatrics and Neonatology, Centre of Child and Adolescent Medicine, Justus Liebig University Giessen, Giessen, Germany; ^3^Institute of Clinical Chemistry and Laboratory Medicine, University Hospital Carl Gustav Carus, Technical University Dresden, Dresden, Germany; ^4^Department of General Paediatrics and Neonatology, Centre of Child and Adolescent Medicine, Justus Liebig University Giessen, Giessen, Germany

**Keywords:** catecholamines, metanephrines, 24-h shifts, work stressor, physicians

## Abstract

Multiple stressors, including 24-h-shifts characterise the working environment of physicians, influencing well-being, health and performance. We aimed to evaluate the effect of the stressor 24-h-shift on the adrenal medullary and sympathoneural system in physicians with the hypothesis that shift work might have different impacts on both systems. Twenty-two physicians collected two 12-h-urine samples (“daytime” and “nighttime”) during a 24-h shift (“on-duty”) and on a free weekend (“off-duty”), respectively. Urinary excretion rates per m^2^ body surface area were assessed for the catecholamines epinephrine, norepinephrine and their respective free O-methylated metabolites metanephrine and normetanephrine by LC-MS/MS-analysis. The stressor provoked differential responses of epinephrine and norepinephrine. Epinephrine excretion rates showed significant increases from off to on duty. The largest proportional change (off-duty to on-duty) for epinephrine was observed for nighttime (205%), the increase for daytime was 84%. An increase in norepinephrine from off to on duty was only visible for nighttime collections. For the catecholamine metabolites, normetanephrine paralleled norepinephrine and exhibited an increase in excretion from off to on duty during nighttime collections of 53% whereas there was no change during daytime collections (3%). In conclusion: Whilst the 24-h-shift-work stressor in physicians activates the sympatho-adrenomedullary system, represented by epinephrine, the sympathoneural response through norepinephrine reflects mainly an ambulatory position during working hours.

## Introduction

Over past decades there has been enlarging awareness about the importance of working conditions and association with work-related “stress” or stressors with adverse health outcomes. Working as a physician is undoubtedly demanding and stressful—physically as well as mentally and emotionally. Numerous scientific studies demonstrate the impact of an environment full of multiple stressors on the well-being of medical doctors in different subspecialties (1–7). One of the main stressors recognised for physicians to be physically challenging and associated with impaired performance witch influences work-life-balance is shift work or being on call (8–12).

Stressor exposure alters activities of different effector systems: besides the hypothalamic-pituitary-adrenocortical system, the sympathoadrenal system is an important mediator of stress-related responses according to separate functions of the adrenomedullary hormonal and sympathetic neuronal systems. Whilst the adrenal medulla is the major source of circulating epinephrine (synonym: adrenaline), stimulation of the sympathetic nervous system leads to discharge of the locally acting neurotransmitter, norepinephrine (synonym: noradrenaline), from sympathetic nerve endings (13).

Responses of the catecholamines, epinephrine and norepinephrine, and their respective O-methylated metabolites metanephrine and normetanephrine (so called metanephrines), are accessible via a non-invasive method, namely timed urine collections: in particular, with liquid chromatography-mass spectrometry tandem mass spectrometry (LC-MS/MS) excretion of both catecholamines and metanephrines can be quantified in 12- or 24-h urine collections.

In the present study, we aimed to evaluate the effect of the stressor “24-h shift” on the adrenal medullary and sympathoneural system in physicians by measuring urinary outputs of catecholamines and metanephrines. As literature tells us that stressors provoke differential responses of components of the autonomic nervous system (14), we hypothesised that the stress of shift work might result in different impacts on both systems and involve an activation mainly of the adrenal medulla.

## Methods

### Participants

Subjects who were eligible for the study included fulltime physicians (occupational level: registrar) of the University Children's Hospital Giessen who took part at regular 24-h weekday shifts (starting with a regular working day on the ward at 8:00 a.m. to 5:00 p.m. with subsequent on-call-duty till 8:00 a.m. the following morning). The study was designed as a prospective crossover trial with each physician completing a 24-h shift (“on duty”) and a 24-h control period on a free Sunday (“off duty”). Age, sex, height and weight were recorded and current medications documented. During the 24-h shift, hours of sleep at nighttime were recorded. Participating physicians provided written informed consent. The institutional review board of the Justus-Liebig-University approved the study (no. 277/11).

### Urine Sample Collection

The probands collected urine samples at two time points: when enduring a 24-h shift at the Children's Hospital (on duty) on a weekday (Monday-Thursday) and on a free and relaxing Sunday at home (off duty). The participants were told to avoid extensive physical activity off duty, with going for a walk at most. Each collection period was divided into two further 12-h time periods: from 8:00 a.m. to 8:00 p.m. (“daytime”) and from 8:00 p.m. to 8:00 a.m. the very next morning (“nighttime”) resulting in four 12-h urine samples per physician. The participants were instructed to start the micturition sampling periods with an emptied bladder, to catch the urine completely on each occasion of micturition, including a final collection before the ending of every 12-h period. For each 12-h sampling period, a new opaque collecting container without preservative was used. Aliquots of all samples were deep frozen (−80°C) within an hour of completion of the 24-h shift or the Monday following the Sunday sampling period. Under these conditions of collection catecholamines and their metabolites have been determined to remain stable even without preservative (15). In order to support completeness of collection, concomitant urinary creatinine values were determined in each 12-h urine sample.

### Urine Analysis

The frozen samples were shipped on dry ice to the Institute of Clinical Chemistry and Laboratory Medicine of the University Hospital Carl Gustav Carus in Dresden/Germany for analysis. Urinary catecholamines epinephrine, norepinephrine and their respective free O-methylated metabolites metanephrine and normetanephrine were measured by liquid chromatography-tandem mass spectrometry (LC-MS/MS) as described previously (15).

Results are presented as urinary outputs per 12-h (nmol/12 h) normalised for body surface area (BSA) and as percentage changes off to on duty. Twenty-four hours urinary outputs (nmol/24-h) of catecholamines and catecholamine metabolites were also calculated in order to allow for comparisons with published literature.

### Statistical Analysis

We based our sample size calculations on a publication from a Dutch research group (16) investigating urine stress hormone secretion from workers under stress conditions and their observed excretion rates (ng/min) and standard deviation at 8 PM sampling time. Assuming a type I error rate of α = 0.05, a statistical power of 80%, and a correlation of 0.2 between paired observations, we estimated a minimal sample size of *n* = 13 for epinephrine and *n* = 17 for norepinephrine. With an estimated dropout rate of 15%, we needed to recruit at least 20 probands to contribute to the study.

Descriptive analysis included calculation of median and interquartile ranges. Wilcoxon signed rank test was applied to test for differences in urinary parameters' concentrations between on duty and off duty, according to hours of sleep (categorised into ≤4-h and >4-h) and males and females. Differences in changes off to on duty include figures showing geometric means and 95% confidence intervals (95%CIs). The use of geometric means is appropriate for non-normally distributed data and allows presentation of CIs for variance for graphical display. Statistical analyses were performed using GraphPad Prism 6 and Stata Release 15 (College Station, TX: StataCorp LLC).

## Results

### Description of Study Cohort

For our study, 22 physicians (12 males and 10 females)—all of them non-smokers—provided agreement to participate. Due to intake of bisoprolol and insulin (drugs known to influence catecholamine levels) two males were excluded from analysis. Other medications used were levothyroxine (two females) and cetirizine dihydrochloride (one male). All but one female took hormonal contraception measures. The participants' characteristics, sleeping features on duty nighttime and data dealing with urine sampling are presented in Table 1.

**Table 1:** Probands' characteristics, sleeping features and descriptive urine sample data.

	**All**	**Females**	**Males**
**Demographics**			
*N*	20	10	10
Age (years)	31.0 [28.3–37.8]	29.5 [27.8–32.3]	32.5 [30.0–37.8]
Body surface area (BSA) (m2)	1.8 [1.4–2.5]	1.6 [1.5–1.7]^*^	1.9 [1.8–2.3]
Hours of sleep	3.9 [3.1–4.7]	3.8 [3.3–4.4]	3.9 [3.0–5.1]
**Urine sample volume (L)**			
On duty daytime	0.80 [0.52–1.13]	0.79 [0.37–0.85]	0.94 [0.66–1.33]
On duty nighttime	1.12 [0.41–1.48]	1.01 [0.38–1.44]	1.12 [0.41–1.48]
Off duty daytime	1.29 [0.82–1.60]	1.53 [1.00–1.60]	0.95 [0.50–1.56]
Off duty nighttime	0.79 [0.69–1.40]	0.79 [0.59–1.66]	0.82 [0.70–1.24]

*All results except proband numbers are presented as median and interquartile ranges*.

* *p = 0.0016*.

### Urine Analysis

Urinary creatinine values were not different on duty daytime (median 7.2 mmol/L, interquartile range 5.7–14.2 mmol/L) compared to off duty daytime (median 5.9 mmol/L, interquartile range 3.9–8.5 mmol/L) and on duty nighttime (median 7.2 mmol/L, interquartile range 5.3–14.2 mmol/L) compared to off duty nighttime (median 6.0 mmol/L, interquartile range 5.4–10.3 mmol/L). All creatinine values were within normal limits. Completeness of urine collections could therefore be assumed.

The — in the interest of comparability — calculated 24-h concentrations of catecholamines and their metabolites were comparable to those previously published (15, 17): For epinephrine, the median [interquartile range] concentration (nmol/24-h) off duty was 26.8 [18.9–49.0] and on duty 62.9 [45.8–85.5]; norepinephrine showed a median off duty of 139.7 [94.2–201.9] and on duty 152.8 [108.4–243.6]; the median [interquartile range] of metanephrine was 117.2 [81.0–194.8] off duty and 137.0 [121.0–194.3] on duty, for normetanephrine 124.0 [46.5–180.5] off and 135.9 [103.2–188.8] on duty.

The 12-h excretion rates of epinephrine showed significant increases from off to on duty collection periods (Table 2). In contrast, an increase in norepinephrine excretion from off to on duty was only visible for nighttime collections. For the metabolites, differences for urinary outputs of normetanephrine showed parallel changes to outputs of norepinephrine, whereas metanephrine showed an increase from off to on duty for nighttime collections only.

**Table 2:** Urinary excretion (nmol/12-h/BSA m^2^) of catecholamines and free metanephrines during on and off duty, nighttime, and daytime.

	**Off duty**	**On duty**	***P*-value**
Epinephrine nighttime	3.7 [2.2–8.2]	11.9 [7.2–23]	0.001
Epinephrine daytime	10.1 [8.0–15.1]	20.1 [15.7–26.8]	0.002
Norepinephrine nighttime	24.7 [19.6–30.4]	39.9 [23.7–63.6]	0.026
Norepinephrine daytime	45.5 [33.8–58.4]	40.6 [33.0–60.4]	1.000
Metanephrine nighttime	27.8 [20.6–38.4]	38.0 [27.7–56.0]	0.038
Metanephrine daytime	29.8 [26.6–44.3]	37.2 [29.5–45.0]	0.982
Normetanephrine nighttime	23.4 [19.8–28.1]	39.6 [22.5–54.6]	0.006
Normetanephrine daytime	34.3 [27.0–45.7]	36.0 [27.2–52.7]	1.000

*Results are shown as medians and interquartiles. P-values derived from paired Wilcoxon signed rank sum test with Bonferroni correction for multiple testing (number of tests = 4).*.

**Figure 1:**
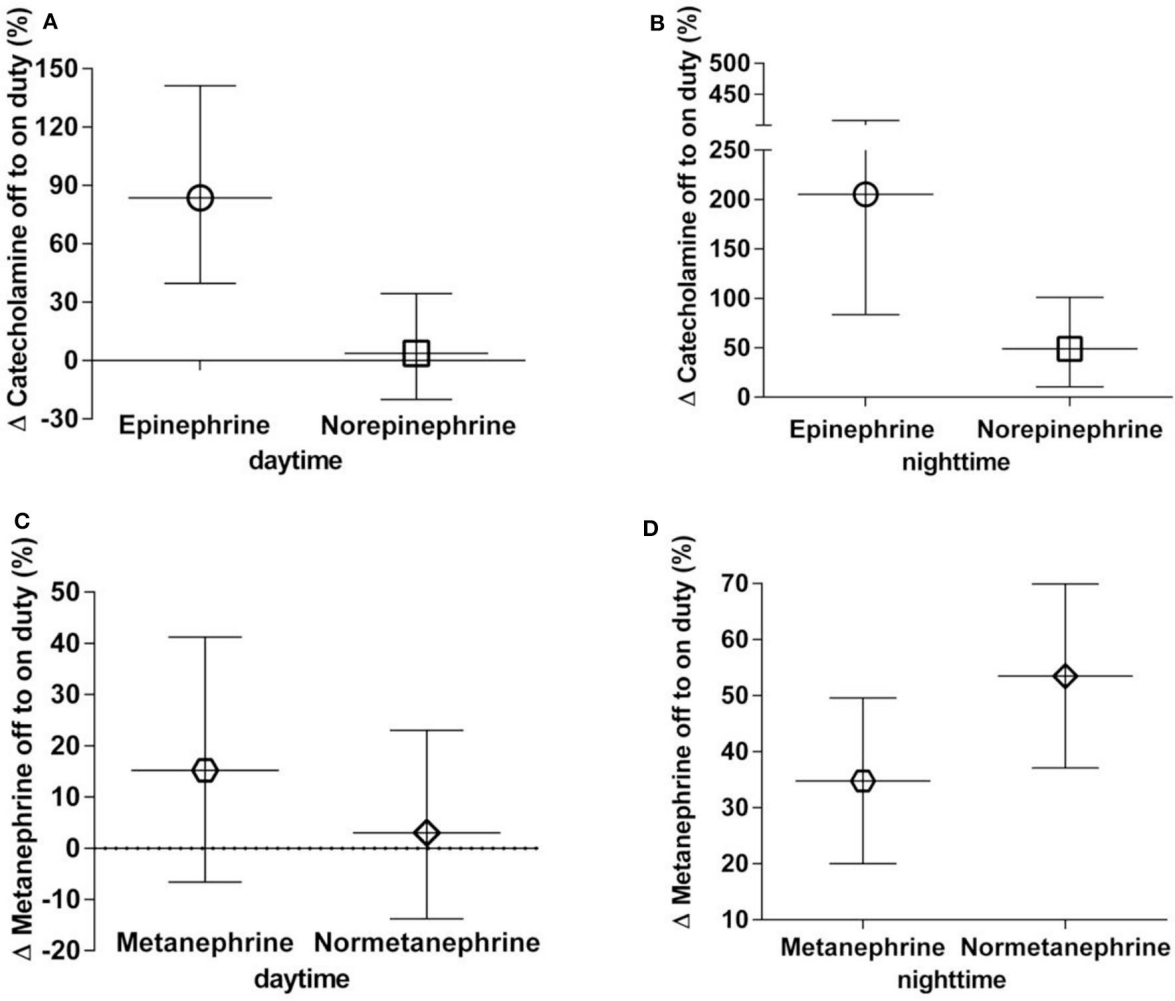
**(A–D)** Percent off to on duty changes in urinary excretion rates of catecholamines **(A,B)** and metanephrines **(C,D)**, daytime and nighttime, respectively. Data are shown as geometric means with 95% confidence intervals.

From assessments of off to on duty differences (delta), the largest proportional changes were observed for epinephrine (nighttime 205% increase, daytime 84% increase) (Figure 1). Norepinephrine showed a 49% increase in excretion from off to on duty for nighttime collections compared to a mere 4% difference for daytime collections. For the O-methylated catecholamine metabolites, normetanephrine exhibited a increase in excretion from off to on duty during nighttime collections of 53%. No change (3%) was seen during daytime collections.

Dividing the whole group of physicians in a female and a male group and comparing the parameters' percent changes off to on duty daytime and nighttime revealed no differences between both sexes.

**Figure 2:**
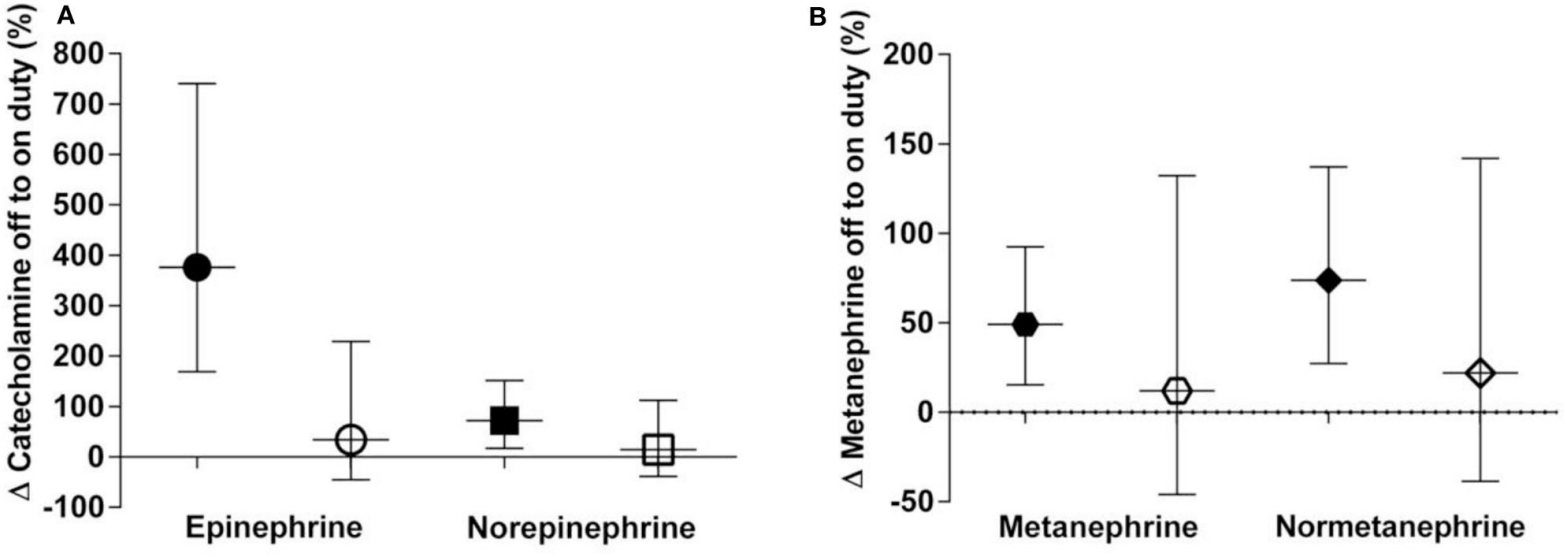
**(A,B)** Percent off to on duty changes categorised into ≤4 h of sleep (black symbols) and >4 h of sleep (clear symbols) in urinary excretion rates of catecholamines **(A)** and metanephrines **(B)**. Data are shown as geometric means with 95% confidence intervals.

During nighttime on duty, 13 participants got four or less hours of sleep, seven participants slept more than 4 h. Applying Wilcoxon signed rank test revealed distinctly higher epinephrine excretion rates for the group with ≤4 h of sleep compared to the group with >4 h of sleep (*p* = 0.048 after correction for multiple comparisons) (Figure 2). No difference could be noticed for norepinephrine or the metanephrines.

## Discussion

Research studies involving stress and catecholamines are often conducted under laboratory conditions exposing probands to one defined stimulus or stressor at a time. Such study designs often lack lab-to life generalisability. Our study design falls into the category of a “naturalistic study” of work stress involving a mixture of different, intermingled stressors: on duty physicians are exposed, inter alia, to long ambulatory hours, sleep deprivation and mental/emotional challenges. Use of urine rather than blood collections provides the opportunity to remain non-invasive, to cover long durations of stressor exposure, and offers a feasible study protocol for physicians during on and off duty working hours where repeated blood sampling would be impractical.

In response to the 24-h-shift we observed divergent results for the catecholamines, epinephrine and norepinephrine, as well as their respective O-methylated metabolites. Whilst urinary excretion of epinephrine consistently increased during both daytime and nighttime off duty to on duty hours, the same effect for norepinephrine was only apparent for nighttime off duty to on duty hours. We interpret these findings as follows: The higher rates of epinephrine excretion from off to on duty truly reflect “stress,” presumably mental/emotional stress that persists in both day and night on duty work hours. In contrast, the increase in norepinephrine excretion observed only for off to on duty nighttime hours more likely reflects the switch from supine rest during the nighttime off duty period to the more ambulatory hours on duty. Lack of increase in norepinephrine excretion from off to on duty for daytime hours likely reflects similar upright posture of probands, who were likely “on their feet” (or at least in a non-supine position) during both off and on duty daytime hours. These data therefore support a specific impact of work stress in physicians on adrenal medullary release of epinephrine.

That stimuli can provoke differential sympathoneural and sympathoadrenal responses is well-established. Robertson et al. (18) established in the 1970's that different interventions (e.g., upright posture, exercising, cold pressure test, and syncope) resulted in divergent responses of plasma catecholamines. They found that orthostasis and treadmill exercise were especially potent stimuli for norepinephrine, whereas the cold pressure test and syncope resulted in marked increases in plasma epinephrine. For mental stress, specifically the Stroop colour-word conflict test, Åkerstedt et al. (19) noted increased urinary excretion of epinephrine but not norepinephrine. Similar findings were reported by Halbrügge et al. (20): an increase in plasma norepinephrine concentrations provoked by orthostasis and an increase in epinephrine by a mental stressor. Tulen et al. (21) also reported posture-related differential effects in plasma catecholamines.

In general, stressor responses display a certain specificity: Sympathoneural noradrenergic outflows play key roles in redistribution of blood volume, blood delivery to the brain and other organs and regulation of blood pressure and are associated with active escape or avoidance; the adrenalmedullary adrenergic system, however, responds to more global or metabolic threats and is also activated by passive, immobile fear (14, 22). The results of our study are thus in line with current concepts concerning divergent effects of stressors on catecholamines and extend this understanding beyond laboratory-interventions to “real-life-conditions”.

Our additional findings that sleep or sleep duration on duty correlated predominantly with the percent change in urinary epinephrine excretion also supports catecholamine response specificity; specifically, the more hours of sleep, the smaller the increase in urinary epinephrine excretion from off to on duty. Being awake during the night on duty does not necessarily translate to getting up for physicians as mere phone calls without physical patient contact are common. An equally large percentage change in norepinephrine—similar to epinephrine—with sleeplessness was therefore not expected. Dodt et al. (23) conducted a laboratory study in healthy men that investigated plasma catecholamine concentrations during nocturnal sleep and periods of wakefulness including standing up at the end of the experiment. The group postulated that awakening itself already enhances plasma epinephrine levels whereas subsequent orthostasis activates most prominently norepinephrine.

What about the metanephrines and their response to the shift work stressor? Metanephrine and normetanephrine are extraneuronal O-methylated metabolites of epinephrine and norepinephrine. However, while up to 76% of normetanephrine is produced after extraneuronal uptake and metabolism of norepinephrine released by sympathetic nerves, more than 90% of metanephrine is produced by metabolism of epinephrine within adrenalmedullary chromaffin cells by a process that is independent of catecholamine release (24, 25). Thus, metanephrine is a poor indicator of adrenal medullary epinephrine release, which explains why percent changes in urinary outputs of metanephrine with off to on duty hours were much lower than changes in urinary outputs of epinephrine. In contrast, the percent change in urinary output of normetanephrine during off to on duty nighttime hours paralleled the changes in norepinephrine, which is consistent with dependence of this metabolite on its production from norepinephrine after release from sympathetic nerves. Therefore, while normetanephrine echoed the sympathoneural response to the shift work stressor, or rather more likely upright posture, metanephrine did not.

One limitation of the study involves lack of control for food and beverage intake, as dietary restrictions most likely would have resulted in a fewer number of probands. Caffeine for example is well-known for stimulating release of catecholamines and potentially impacting stressor reactivity (26, 27), whilst a dose dependency is less clear (27, 28). Thus, we cannot exclude an influence of caffeine on off to on duty increases in urinary excretion of epinephrine. Nevertheless, one might assume that the coffee- (or tea-) drinking physician might consume this beverage both on and off duty, at least during the daytime hours. Due to the cross over character of the present study each individual serves as their own control, so that a massive influence of stimulating beverages seems unlikely. Another issue concerns potential impacts of food and fasting: Consumption of meals results in regionalised changes in sympathoneural outflow and decreased adrenalmedullary release of epinephrine, manifest by increased plasma norepinephrine and decreased plasma epinephrine (29, 30). There were no instructions when to eat or not to eat in the study protocol. Nevertheless, it seems unlikely that off to on duty increases in urinary outputs of epinephrine might reflect an impact of meals, particularly for nighttime off to on duty hours.

Although influences of sex on plasma and urinary epinephrine and metanephrine are established (15, 24), we noted no difference between females and males in percent changes of these parameters. Therefore, an equal catecholamine/metanephrine reactivity toward the stressor can be assumed. The majority of female physicians of the study used hormonal contraception measures, and contraceptives might affect “female” catecholamine levels (31, 32). Nevertheless, via contraceptive intake, a certain cycle conformity was provided; and since our study involved intraindividual comparisons it is unlikely that the “female” stressor response pattern was influenced by hormonal contraceptive measures.

Elevations in circulating catecholamines have time and again been connected with health conditions, especially high blood pressure (33–35). The brief “snapshot” of our study with limited proband numbers in a single centre, however, does not allow at this time any generalised speculation about possible health consequences due to the 24-h-work-stressor. In conclusion, we establish that a 24-h-shift-work stressor in physicians is associated with activation of the sympatho-adrenalmedullary system, manifest by increased epinephrine excretion on duty. The sympathoneural response (norepinephrine) is restricted nighttime on duty, likely reflecting a change from supine sleep to ambulatory work hours. Female and male physicians respond similarly to the stressor.

## Data Availability Statement

The raw data supporting the conclusions of this article will be made available by the authors, without undue reservation.

## Ethics Statement

The studies involving human participants were reviewed and approved by Justus-Liebig-University. The patients/participants provided their written informed consent to participate in this study.

## Author Contributions

CB, SW, and K-PZ contributed substantially to the conception and design of the work. CB, GS, MP, and GE are responsible for the analysis and interpretation of data for the work. CB drafted the manuscript. All authors are responsible for data acquisition for the work, revised the manuscript critically for important intellectual content, provided approval for publication of the content, and agreed to be accountable for all aspects of the work in ensuring that questions related to the accuracy or integrity of any part of the work are appropriately investigated and resolved.

## Conflict of Interest

The authors declare that the research was conducted in the absence of any commercial or financial relationships that could be construed as a potential conflict of interest.
